# Gender differences in guilt aversion in Korea and the United Kingdom

**DOI:** 10.1038/s41598-022-12163-y

**Published:** 2022-05-17

**Authors:** Tsuyoshi Nihonsugi, Toshiko Tanaka, Masahiko Haruno

**Affiliations:** 1 Faculty of Economics, Osaka University of Economics, Osaka, Japan; 2 Center for Information and Neural Networks (CiNet), National Institute of Information and Communications Technology (NICT), Suita, Japan; 3 Graduate School of Frontier Biosciences, Osaka University, Suita, Japan

**Keywords:** Neuroscience, Psychology

## Abstract

Guilt aversion, which describes the tendency to reduce the discrepancy between a partner’s expectation and his/her actual outcome, is a key driving force for cooperation in both the East and West. A recent study based on functional magnetic resonance imaging and online behavioral experiments reported that men show stronger guilt aversion than women and also suggested that men’s predominance in guilt aversion arises from stronger sensitivity to social norms. However, since the participants of that study were all Japanese, it remains unaddressed how common the gender difference in guilt aversion is. Here, we conducted online behavioral studies on people from Korea and the UK (Korea; *n* = 294, UK; *n* = 347) using the same trust game. We confirmed that men exhibit stronger guilt aversion than women in both countries. Furthermore, consistent with the Japanese study, our Lasso regression analysis for UK participants revealed that Big Five Conscientiousness (rule-based decision) correlated with guilt aversion in men. In contrast, guilt aversion in Korean men correlated with Big Five Neuroticism. Thus, our results suggest that gender differences in guilt aversion are universal but the underlying cognitive processes may be influenced by cultural differences.

## Introduction

Previous research has indicated gender differences in socioeconomic behaviors such as occupational choice^1–4^, judicial rulings^5,6^, medical practice patterns^7^, and educational decisions^8^. It is often hypothesized that these differences are caused by different social preferences between genders. Indeed, a large body of evidence has reported gender differences in social preferences (for example, trust, reciprocity, altruistic, inequality averse and competitive preference; for a review, see^9^).

Guilt aversion is a form of social preference and is an important factor of cooperation in living up to the perceived expectations of others, as people suffer from guilt when they disappoint others^10–13^ (see “Materials and Methods” for a more detailed definition). In psychology, guilt is divided into two categories, *guilt from harm* and *guilt from norm violation*^14,15^. Since guilt aversion theory (belief-dependent guilt) primarily references the awareness of having caused unjustified harm to others, *guilt from harm* is the core concept of this theory. As early seminal study, Battigalli & Dufwenberg^12^ developed a general model of guilt aversion as belief-dependent motivation, which many experimental studies support^11,16^. One study^17^ proposed belief-dependent preference as a combination of guilt aversion (belief-dependent guilt) and intention-based reciprocity and elicited the trustees’ belief-dependent preferences from their answers to a structured questionnaire. The study demonstrated that guilt sensitivity is a dominant psychological motivation in a trust game. Another study^18^ showed that matching more guilt-averse trustees leads to higher trust and longer cooperative paths in a repeated trust game, indicating that guilt sensitivity plays a significant role in repeated interactions. That study also classified trustees according to psychological types of guilt averse, reciprocal, selfish, or unclassified (include inequity aversion), finding that guilt-averse players were the majority and inequity-averse players were less represented. Notably, many experimental studies have demonstrated that belief-dependent guilt is a predominant motivator for trustees in a trust game (for a broader discussion, see^19^). Additionally, both laboratory and field experiments have supported guilt aversion theory for populations in both the East and West (e.g., East^20–22^; West^11,17,18,23–30^).

Although guilt aversion depends on one’s own beliefs about the underlying intention of others, a comparison of behavioral outcomes between the self and other is also important for social decision making^31^. Inequity aversion^32^ is the most perceived form of outcome-based decision making, which is defined as the propensity to avoid an imbalance between outcomes for the self and the other. Additionally, empirical evidence has revealed gender differences in inequity aversion^9^. However, only a limited number of reports have examined gender differences in guilt aversion.

Some psychological studies have indicated that women are more empathetic^33,34^ and prone to guilt and shame^35,36^, as assessed by scenario-based measures, while men feel more guilt^36^, as assessed by the Guilt Inventory^37,38^. A previous meta-analysis^39^ regarding guilt and shame found that both had small gender differences, and gender differences in shame and guilt were only significant for white populations. Another study^40^ demonstrated that boys’ guilt reactions are more directly based on cognition and reasoning than girls’ ones, although girls are more guilt prone, suggesting that boys and girls may reference different aspects of guilt. Altogether, these previous studies suggest that gender differences in guilt sensitivity are influenced by cultural differences and reflect distinct cognitive strategies used by men and women.

Guilt aversion requires the ability to assess another individual’s expectations, and it is directly related to the other’s disappointment (i.e., empathy or theory of mind^41^). Moreover, guilt aversion represents psychological pressure and is directly related to the sensitivity of negative emotions (i.e., the sensitivity of negative emotions^10^). At the same time, guilt aversion is a normative behavior elicited by experience (i.e., rule-based decision making^42^). Hence, if there are gender differences in guilt aversion, these three potential cognitive strategies—empathetic consideration, the sensitivity of negative emotion, and rule-based decision making—may contribute to such differences.

Most experimental studies have predominantly found that women exhibit trustworthiness (reciprocate) at least as much, or even more, than men (for a review, see^9^). A recent experimental economics study^43^ investigated gender differences in the motives for promise-keeping, which are closely related to trustworthiness, conducting a modified dictator game including a communication stage. This study found that the primary motives for both men’s and women’s promise-keeping are social norms (to fulfill a commitment) and others’ expectations (guilt aversion). The research revealed that although women keep promises more often than men, there are no gender differences in the underlying motives for promise-keeping. Conversely, other experimental research^44^ reported a lower, though not significant, proportion of women who keep promises than men, indicating that men may be more guilt averse. However, these studies analyzed gender differences in terms of promise-keeping and the motivations underlying it, and do not estimate guilt sensitivity and investigate gender differences in the estimated guilt sensitivity; thus, potential gender differences in guilt aversion remain poorly understood.

In our recent study^21^, functional magnetic resonance imaging and online behavioral experiments (Japanese participants; *n* = 4723; 2737 women) were performed to directly estimate guilt sensitivity from participants’ choices in 45 trials. Comparing men and women while controlling for the confounding effects of participants’ socioeconomic status, that study demonstrated that men have stronger guilt aversion than women. It also revealed that the Big Five conscientiousness (rule-based decision making) correlates only with guilt aversion in men, while Big Five agreeableness (empathetic consideration) correlates with guilt aversion in both genders. The study also revealed that the prefrontal network related to social norms and self-control plays a key role in guilt-based prosocial behavior in men. Overall, then, the study suggests that the consideration of social norms plays a key role in men’s predominance in guilt aversion. However, the study was based on an exclusively Japanese population. To our knowledge, no study has examined cross-country tests for gender differences in guilt aversion and its cognitive underpinning.

Here, to address this issue, we investigated gender differences in guilt aversion and the cognitive underpinnings by conducting online experiments on people living in Korea and the United Kingdom (UK) using the same task as the Japanese study, which was designed to study guilt aversion as well as inequity aversion. The two countries have relatively the same economic status (according to the World Economic Outlook Database^45^, the 2020 per-capita GDP in the UK was US$44,153, while that of Korea was US$44,750).

We also collected demographic information and Big Five scores. The Big Five personality traits^46^, widely known as a measure of individual personality, are based on five dimensions: neuroticism, extraversion, openness, agreeableness, and conscientiousness. Agreeableness is characterized by the understanding of others’ emotions, intentions, and mental states; neuroticism is characterized by negative emotion and includes various traits such as anxiety, anger, depression, and shame; and conscientiousness is characterized by rule-based regulation and self-discipline. Women consistently score higher than men on agreeableness and neuroticism across countries, whereas gender differences in conscientiousness are not consistent across cultures, and no significant gender difference has typically been found^47^. These findings suggest that if gender differences do exist in guilt-aversion behavior, there may also be cultural differences in the underlying cognitive strategies.

## Materials and methods

### Participants

The number of participants was determined based on the suggestion that at least 10 events should be observed for every explanatory variable entered into the regression model^48^. Since our Lasso and linear regression models include 17 explanatory variables, our recruitment goal was a sample size of more than 170 individuals. The participants were recruited from the registrants of an Internet research service company (NTT Com Research, Inc.). We analyzed the data of 294 participants in Korea (175 women) and 347 participants in the UK (190 women) (for more descriptive statistics, see Supplementary Table S1) who followed the task instructions correctly. Given a target of 80% statistical power and 95% probability of detecting an effect of medium size (Cohen’s ${f}^{2}$ = 0.15), this power analysis requires a sample size of 128 in a linear regression model, which our sample size met. All research methods were carried out in accordance with the Code of Ethics of Japanese Psychological Association (3rd) and Ethics Checklist of Japanese Behavioral Sciences Team (BEST), and all the experimental procedures of this study were approved by the ethics committee of the National Institute of Information and Communications Technology, and were checked for the handling of the personal data. Informed consent was obtained from all participants. If the participants under the age of 18, informed consent was obtained from their parents or legal guardian.

### Task

**Figure 1:**
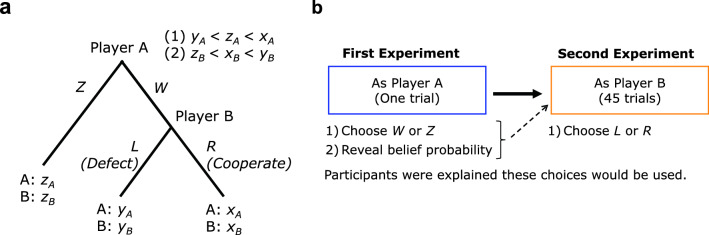
Design of the trust game. (**a**) First, Player A chooses *W* or *Z* and reveals the belief probability (${\tau }_{A}$) that Player B will choose *R* (*Cooperate*). If Player A chooses *Z*, then Players A and B receive ${z}_{A}$ and ${z}_{B}$, respectively. If Player A chooses *W* (i.e., he/she trusts Player B), then with the knowledge of Player A’s belief probability, Player B decides whether to *L* (*Defect*) or *R* (*Cooperate*). If Player B chooses *L*, Players A and B receive ${y}_{A}$ and ${y}_{B}$, respectively; if Player B chooses *R*, Players A and B receive ${x}_{A}$ and ${x}_{B}$, respectively. (**b**) An illustration of the complete experimental paradigm. In the first experiment, which contained one trial, each participant (as Player A) chooses *W* or *Z* and reveals his/her belief probability that Player B will choose *R*. In the second experiment, which contained 45 trials, each participant (as Player B) chooses *L* or *R*. Participants were told that the other participant (player A) differed for each trial.

In this task, adapted from previous experimental studies^11,49^, two participants were paired as Players A and B (see Fig. 1a). First, Player A was asked to choose between playing *W* or *Z* and reveal his/her belief about ${\tau }_{A}$ (0%–100%), i.e., the probability that Player B will choose *R* (i.e., *Cooperate*). In other words, ${\tau }_{A}$ represents Player A’s level of trust in Player B. If Player A choose *Z*, then Players A and B receive payments ${z}_{A}$ and ${z}_{B}$, respectively. If Player A chooses *W*, then knowing Player A’s belief probability, Player B is asked to choose between playing *L* (i.e., *Defect*) or *R*. If Player B chooses *L*, then Player A receives ${y}_{A}$ and Player B receives ${y}_{B}$, whereas if Player B chooses *R*, then the two players receive ${x}_{A}$ and ${x}_{B}$, respectively. For convenience, *L* and *R* in Fig. 1a are denoted as *Defect* and *Cooperate*, respectively. Note that although the expressions *L* and *R* represent the terms *Defect* and *Cooperate*, respectively, these terms were not used in our experiment.

Figure 1a presents two important conditions regarding the payments: if (1) ${y}_{A}<{z}_{A}<{x}_{A}$, then Player A signals trust (cooperation) to Player B when Player A chooses *W*; if (2) ${z}_{B}<{x}_{B}<{y}_{B}$, then Player B feels guilt upon disappointing Player A relative to Player A’s belief in what Player A will receive. These two conditions are necessary for guilt aversion theory. Additionally, if Player B is rational and selfish, then Player B chooses *Defect* (*L*). Player A, anticipating this action, will choose *Z* and produce the allocation of the money payoffs ${z}_{A}$ and ${z}_{B}$. This online trust game was also used in our previous study^15^.

### Procedure

In the trust game, the participants were involved in two consecutive behavioral measurements (Fig. 1b; see also the instructions in the Supplemental Information). Before the first experiment, online participants read the rules of the trust game and the procedure. In the first experiment, each participant played the trust game as Player A (i.e., choose *W* or *Z* and reveal belief probability ${\tau }_{A}$) and experienced one trial. The participants knew that these choices would be used when Player B was asked to make a choice in the second experiment. Note that Ellingsen et al.^49^ use a similar approach, but in their experiment, Player A reveals his/her belief without knowing that the belief will be revealed to Player B before Player B’s choice. In reality, the ${\tau }_{A}$ that Player A revealed was not used when Player B made decisions. Further, the ${\tau }_{A}$ of the 45 trials was predetermined by the experimenter. Neither Player A nor B was informed of this during the experiment. This approach looks like deception. There is a strong norm against the use of deception in experimental economics; however, according to Charness et al.^50^, precisely what constitutes deception is unclear, and they argued against a blanket ban, calling for a more nuanced view. Specifically, they mentioned that there is support for deception when the data are important, especially if this will not greatly impact any subject pool in the future. We believe that our experiment met this required standard. Participants were instructed that Player B was another participant who would take part in the second experiment. However, Player A was not informed of Player B’s identity. The second experiment comprised 45 trials, with each participant involved in performing Player B’s role (i.e., choose *Defect* or *Cooperate*) conditional on the matched Player A choosing *W* and without knowing Player A’s actual choice. The participants were also told that Player A will differ for each trial and that the pairings will be anonymous. The sequence of the trials was randomized across participants, and no feedback was provided to the participants during the experiment. Additionally, each participant completed the personality trait tests (the Big Five Inventory-10 for Korea^51^ and for the UK^52^; see also the personality questionnaire in the Supplemental Information). Big Five Inventory-10 is a 10-item short version of the Big Five Inventory-44^53^ and provides a measure of the Big Five for contexts in which a participant’s time is severely limited.

For their participation, participants were paid in cashable points, proportional to the number of payoffs earned during the experiment (equivalent to US$3–5). The participants were also informed that the final payoff would be the sum of the decisions made as Players A and B. For the second experiment, the participants were told that the earnings for Player B would be the sum of the actual outcome of the 45 trials (instead of randomly selecting only one trial).

### Guilt aversion and inequity aversion

We illustrate the guilt aversion model using the guilt sensitivity introduced by previous theoretical and experimental studies^11,12^. In line with Attanasi et al.^54^, we first assume that guilt sensitivity is role-dependent, and only the second mover can be affected by guilt. In short, because of role asymmetry in the game, Player B’s guilt sensitivity can be positive while that of Player A is null. Note that guilt sensitivity elicited in the trust game by guilt aversion theory is fundamentally related to the Test of Self-Conscious Affect-3 (TOSCA-3) and the Guilt and Shame Proneness Scale (GASP), which is a common measure of guilt sensitivity in psychology but is unrelated to shame^55,56^.

A guilt-averse player suffers from guilt to the extent he/she believes he/she hurts others relative to what they believe they will get. Therefore, individuals are motivated by their own beliefs. The guilt aversion model includes social pressure on Player B if the profile (*W*, *Defect*) is played (see Fig. 1a). Let ${\tau }_{A}$ denote Player A’s belief about the probability (0% to 100%) that Player B will choose *Cooperate*. Player B is assumed to believe that if Player A chooses *W*, then Player A believes that he/she will get a return of ${\tau }_{A}\cdot {x}_{A}+(1-{\tau }_{A})\cdot {y}_{A}$, as the setting of Player A’s payoff is ${y}_{A}<{z}_{A}<{x}_{A}$. The difference,${\{\tau }_{A}\cdot {x}_{A}+\left(1-{\tau }_{A}\right)\cdot {y}_{A}\}-{y}_{A}={\tau }_{A}({x}_{A}-{y}_{A})$, which is non-negative in our settings, can measure how much Player B believes he/she hurts Player A relative to what Player A believes he/she will get if Player B chooses *Defect*. In other words, the difference ${\tau }_{A}({x}_{A}-{y}_{A})$ is the amount of guilt that Player B experiences. Let us assume that parameter ${\gamma }_{B}$ measures Player B’s sensitivity to guilt. A player is guilt-averse and will *Cooperate* if ${y}_{B}-{\gamma }_{B}\cdot {\tau }_{A}({x}_{A}-{y}_{A})<{x}_{B}$.

By contrast, an individual is known to have the preference for fairness and resistance to incidental inequalities^32^. Therefore, the inequity aversion model assumes that their utility decreases when the allocation of monetary payoffs differs. Thus, if an inequity-averse player suffers from inequity, he/she make decisions so as to minimize inequity in outcomes. Notably, the advantageous inequity (receiving a larger reward than others) in Fehr and Schmidt’s inequity aversion model is also referred to as “guilt.” However, this outcome-based “guilt” and the intension-based “guilt” that we treat in guilt aversion are completely different.

Notably, in the standard inequity aversion model^32^, the utility function (${u}_{B}$) for Player B is as follows:

$$ u_{B} = \left\{ {\begin{array}{*{20}l} {x_{B} - \varphi_{B} max\left\{ {x_{A} - x_{B} , 0} \right\} - \omega_{B} max\left\{ {x_{B} - x_{A} , 0} \right\}} \hfill & { if\; the \;profile \left( {W, \;Cooperate} \right)} \hfill \\ {y_{B} - \varphi_{B} max\left\{ {y_{A} - y_{B} , 0} \right\} - \omega_{B} max\left\{ {y_{B} - y_{A} , 0} \right\}} \hfill & { if \;the\; profile \left( {\begin{array}{*{20}c} {W, \;Defect} \\ \end{array} } \right)} \hfill \\ \end{array} } \right., $$

The second term measures the utility loss from disadvantageous inequality, while the third term measures the loss from advantageous inequality. ${\varphi }_{B}$ and ${\omega }_{B}$ are constants measuring Player B’s sensitivity to disadvantageous inequity and advantageous inequity, respectively. In line with the role-dependent assumption in the guilt aversion model, we assume that also ${\varphi }_{B}$ and ${\omega }_{B}$ are role-dependent, i.e., only the second mover can be affected by disadvantageous inequity and advantageous inequity.

On the other hand, the utility function (${u}_{B}$) of the inequity model of absolute difference^21^ for Player B is as follows:

$$ u_{B} = \left\{ {\begin{array}{*{20}l} {x_{B} - \alpha_{B} \left| {x_{A} - x_{B} } \right|} \hfill & { if\; the \;profile \left( {W, Cooperate} \right)} \hfill \\ {y_{B} - \alpha_{B} \left| {y_{A} - y_{B} } \right|} \hfill & { if \;the \;profile \left( {\begin{array}{*{20}c} {W, Defect} \\ \end{array} } \right),} \hfill \\ \end{array} } \right. $$

where ${\alpha }_{B}$ is a constant measuring Player B’s sensitivity to inequity. This model does not divide inequity into disadvantageous inequality and advantageous inequality. As mentioned in the Results section, using the Bayesian information criterion (BIC), we found that the inequity model of absolute difference was superior than the standard inequity aversion model. Based on this finding, we judged the absolute difference model for inequity to be a plausible one for the current task.

We further integrated guilt aversion and inequity aversion (absolute inequity) into ${u}_{B}$ for Player B as follows:

$$ u_{B} = \left\{ {\begin{array}{*{20}l} {x_{B} - \alpha_{B} \left| {x_{A} - x_{B} } \right|} \hfill & {if \,  the \, profile \,  \left( {W, Cooperate} \right)} \hfill \\ {y_{B} - \gamma_{B} \cdot \tau_{A} \cdot \left( {x_{A} - y_{A} } \right) - \alpha_{B} \left| {y_{A} - y_{B} } \right|} \hfill & {if \,  the \,  profile  \, \left( {\begin{array}{*{20}c} {W, Defect} \\ \end{array} } \right),} \hfill \\ \end{array} } \right. $$

A narrowly self-interested agent is given the special case ${{\gamma }_{B}=\alpha }_{B}=0$. In our game, the players choose between binary actions that yield two different monetary payoff allocations: $X=\left({x}_{A}, {x}_{B}\right)$ and $Y=\left({y}_{A}, {y}_{B}\right)$. The utilities of these allocations are given by the aforementioned formula, yielding ${u}_{B}(X)$ and ${u}_{B}(Y)$.

### Logistic regression of the behavioral data

Based on the logistic model of stochastic choice, we estimated three separate components (i.e., monetary self-interest, guilt, and inequity) for each participant. The probability that Player B chooses *Cooperate* can be expressed as ${P}_{B,Cooperate}=1/1+{e}^{{-\{u}_{B}\left(X\right)-{u}_{B}\left(Y\right)\}}$. We then used logistic regression as follows:

$$logit({P}_{B,Cooperate})={\beta }_{0}+{\beta }_{1}{Reward}_{t}+{\beta }_{2}{Guilt}_{t}+{\beta }_{3}{Inequity}_{t}$$

where ${Reward}_{t}$ is the reward size, calculated as ${x}_{B}-{y}_{B}$ at time *t*, ${Guilt}_{t}$ is the guilt size calculated as $-\{0-{\tau }_{A}\cdot {(x}_{A}-{y}_{A})$}, and ${Inequity}_{t}$ is the inequity size calculated as $-(\left|{x}_{A}-{x}_{B}\right|-\left|{y}_{A}-{y}_{B}\right|)$. For convenience,${\beta }_{1}$, ${\beta }_{2}$, and ${\beta }_{3}$ are denoted as $\beta (Reward)$, $\beta (Guilt)$, and $\beta (Inequity)$, respectively. To orthogonalize the three explanatory variables, the actual ${\tau }_{A}$ used in the experiments was also set by the experimenter; thus, the ${\tau }_{A}$ that Player A revealed was not used when Player B made a decision. Instead, Player B was asked to make decisions assuming that Player A chose the *In* option; therefore, we set ${\tau }_{A}$ to 60% or higher (Player A is expected to choose the *Out* option when *τ* is small). More specifically, ${\tau }_{A}$ was 60% 7 times, 70% 5 times, 80% 13 times, 90% 11 times, and 100% 9 times. Neither Player A nor B were informed of this condition during the experiments. The correlation coefficients among the three explanatory variables were less than 0.30 and nonsignificant (*P* > 0.05), and the values of guilt and inequity were designed to be orthogonal [correlation coefficient of these two variables was − 0.045 and nonsignificant (*P* = 0.772)] to dissociate the computational processes for guilt aversion and inequity aversion. This task design allowed us to investigate guilt aversion without the influence of inequity aversion. This logistic analysis was also used in our previous study^21^.

The logistic regression was done in R (R Development Core Team, 2008). We used the brglm package to conduct our maximum likelihood estimation with the bias-reduction method^57^.

### Logistic mixed effects regression of the behavioral data

To identify the relationship between participants’ decisions (*Cooperate* or *Defect*) and gender, we performed a logistic mixed effects regression that included random intercepts to control for within-participants variability and regressed the Reward, Guilt, Inequity, Gender (men = 1), and interactions with Gender and Guilt on the participants’ decisions (cooperate = 1) (see also Table 1). Since our study aimed to investigate whether gender differences in guilt-aversion behavior are observed in countries other than Japan, the analysis was divided by country. The logistic mixed effects regression was done using the glmmML function ^58^ of R.

### Lasso regression of the behavioral data

Having confirmed that Reward, Guilt, and Inequity play crucial roles in the current task using a logistic mixed effects regression, we conducted a Lasso regression^59^ to identify the relationship between $\beta (Guilt)$ and gender under controlled social-demographic environments (age, education, and income) and basic personality traits (Big Five Inventory). Moreover, to investigate the cognitive processes potentially underlying gender differences in guilt aversion, we conducted a second Lasso regression that included the interactions of gender with personality trait scores.

**Figure 2:**
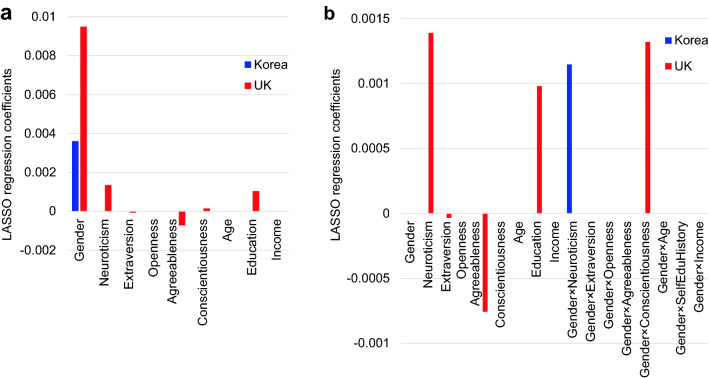
The results of the Lasso regression of gender differences for guilt (except the intercept terms for display purposes). Guilt-aversion behavior ($\beta (Guilt)$) was regressed using Lasso regression, with the following variables as regressors: (**a**) gender, Big Five, and socioeconomic status (age, education, and income); and (**b**) gender, Big Five, socioeconomic status, and the interaction between gender and Big Five and socioeconomic status.

Specifically, to identify the relationship between guilt aversion ($\beta (Guilt)$) and gender, we first performed a Lasso model analysis (target variable: $\beta (Guilt)$) based on the explanatory variables, including gender (men = 1) and the Big Five Inventory-10 and socioeconomic status scores (age, education, and income) for all participants (see also Fig. 2a and Table 2(1)). Next, to identify the cognitive mechanisms specific to gender, we performed a second Lasso model regression (target variable: $\beta (Guilt)$), including interaction terms between the gender variable and the Big Five Inventory-10 and socioeconomic status scores (see also Fig. 2b and Table 2(2)).

Lasso regression^59^ is a variable-selection-and-constraint method that is best used for a large number of predictors, especially when they are highly correlated. The LASSO algorithm constrains or penalizes the absolute size of the regression coefficients so that no coefficients are too large when compared with others. The Lasso procedure not only achieves beneficial shrinkage, but it also ensures that a variable remains in the model with a non-zero coefficient if its incremental predictive value is sufficient for justifying the sacrifice of a degree of freedom. Based on the benefits of Lasso regression, we adapted this method for our behavioral data. Our Lasso regression models were created using the glmnet function of R. The optimal value of the penalty parameter was determined by a ten-fold cross validation using the cv.glmnet function of R (glmnet and cv.glmnet are included in the glmnet package^60^).

We also report the results replacing the Lasso regression analysis with the general linear model (GLM) regression in the Supplementary Information (see Supplementary Tables S2 and S3). We performed the GLM regression with the full model (i.e., 17 variables) as well as with the variables selected in the Lasso regression because the Lasso model is a variable-selection model. Models 1 and 2 in Supplementary Tables S2 and S3 present the full model and variable-selection models, respectively. Note that in the multicollinearity situation, the coefficient estimates of the GLM regression change erratically in response to small changes in the model or the data. In fact, our regression has up to 17 experimental variables, some of which correlate significantly; for instance, Gender and Income in both Korea and the UK (*P* < 0.001), and Income and Education in both Korea and the UK (*P* < 0.001). Therefore, not all of the GLM results are consistent with our Lasso results.

## Results

We conducted an online behavioral study (Korea; *n* = 294 [175 women], UK; *n* = 347 [190 women]) of a trust game that was designed to dissociate the computational processes for guilt aversion and inequity aversion. Our analysis and results begin with the model selection, followed by analyses of the gender differences and the cognitive mechanisms of guilt aversion.

### Model selection

We conducted a statistical analysis of guilt aversion based on Player B’s choices (see “Materials and methods”). For inequity, Fehr and Schmidt’s model^32^, which splits inequity into advantages and disadvantages, is commonly used. However, as we reported previously^21^, BIC between these two models showed that for 3714 out of the 4723 participants (78.6%) the absolute difference for the inequity model was selected. Therefore, we also compared the BIC between Fehr and Schmidt’s model and the absolute difference model in the present study.

We found not only that the absolute difference model had a slightly smaller mean BIC than Fehr and Schmidt’s model for both the Korea and UK participants (Korea, 48.723 vs. 49.680; UK, 50.520 vs. 51.989), but also that for 224 out of the 294 participants (76.2%) in Korea and for 276 out of the 347 participants (79.5%) in the UK, the smallest BIC model was the absolute difference model. Based on these findings, we determined that the absolute difference model for inequity was plausible for the current task.

### Gender differences in guilt aversion

To identify the relationship between participants’ decision making (*Cooperate* or *Defect*) and gender, we performed a mixed effect logistic regression (target variable: cooperate = 1) comprised of fixed effects of Reward, Guilt, Inequity (i.e., absolute difference), Gender (men = 1) and interactions with Gender and Guilt, and random effects of the participants.

We found that the $\beta$ values of Reward, Guilt and Inequity were positive and significant (*P* < 0.001, see Table 1) for both the Korea and UK participants, clarifying that these three components all have a critical effect on behavior in the task. We also found Gender × Guilt to be positive and significant for both Korea and the UK, indicating that men choose *Cooperate* more often than women, in accordance with more guilt. When the participants were guilt averse in our task, they chose *Cooperate*, implying that men exhibited stronger guilt aversion than women in both Korea and the UK.

**Table 1:** Mixed effects logistic regression predicting decisions to *Cooperate* or *Defect.*

Explanatory variable	Dependent variable:$logit({P}_{B,Cooperate})$	
	Korea	UK
Reward	36.202*** (1.337)	28.876*** (1.146)
Guilt	14.430*** (1.561)	13.722*** (1.452)
Inequity	8.582*** (0.623)	11.377*** (0.571)
Gender	− 380.221 (202.048)	− 249.664 (170.550)
Gender × guilt	8.971*** (2.430)	9.203*** (2.127)
Constant	241.972 (139.238)	260.763* (61.158)
McFadden’s R^2^	0.062	0.057
Observations	294	347

Standard errors are in parentheses. All coefficients and standard errors are shown multiplied by 10^3^. Significance: ***P < 0.001; *P < 0.05.

However, the rate of cooperation does not imply guilt sensitivity itself; therefore, we directly assessed guilt sensitivity (i.e., $\beta (Guilt)$) by conducting a Lasso regression to elicit a more robust result regarding gender differences. To identify the relationship between guilt aversion (i.e., $\beta (Guilt)$) and gender under the control of social-demographic environments and basic personality traits, we first performed a Lasso regression (target variable: $\beta (Guilt)$) based on the explanatory variables, including gender (men = 1), Big Five Inventory-10, age, education, and income for all participants.

Figure 2a and Table 2(1) show the Lasso regression results. The coefficient of the gender variable had the highest weight for both the Korean and the UK participants. We also obtained the same result using a general linear model regression (see, Supplementary Table S2, Korea, *P* = 0.0282; UK, *P* = 0.0045). These results are consistent with the mixed logit model results and those of the Japanese study^21^ and suggest that guilt aversion is stronger in men than women in both the East and West.

**Table 2:** Lasso regression of gender differences for guilt.

	Dependent variable:$\beta (Guilt)$			
	(1)		(2)	
Explanatory variable	Korea	UK	Korea	UK
Gender	3.61	9.49	0	0
Neuroticism	0	1.35	0	1.39
Extraversion	0	− 0.07	0	− 0.03
Openness	0	0	0	0
Agreeableness	0	− 0.72	0	− 0.76
Conscientiousness	0	0.15	0	0
Age	0	0	0	0
Education	0	1.04	0	0.98
Income	0	0	0	0
Gender × neuroticism			1.15	0
Gender × extraversion			0	0
Gender × openness			0	0
Gender × agreeableness			0	0
Gender × conscientiousness			0	1.32
Gender × age			0	0
Gender × education			0	0
Gender × income			0	0
(Intercept)	31.35	15.50	30.31	16.40

All scores are Lasso regression coefficients and are shown multiplied by 10^3^.

### Underlying cognitive mechanisms for gender differences in guilt aversion

Next, to identify the cognitive mechanisms for the gender difference, we conducted a second Lasso regression, including interaction terms between the gender (dummy) variable and the Big Five Inventory-10 and socioeconomic status scores. Figure 2b and Table 2(2) present these Lasso regression results. We found that Gender $\times$ Conscientiousness had a high positive weight for the UK population. We also obtained the same result using a GLM regression (see Supplementary Model 2 in Table S2, *P* = 0.0028). This finding shows that for the UK, the stronger guilt aversion in men is attributable to greater use of rule-based (social norm-based) strategies, again consistent with the Japanese study^21^. On the contrary, only Gender $\times$ Neuroticism had a positive weight for the Korean population (a similar result was observed in the GLM regression; see Supplementary Model 2 in Table S2, *P* = 0.0180). The correlation coefficient between Neuroticism and Conscientiousness for Korea was quite small (correlation coefficient; -0.15; see also Supplementary Fig. S1, which presents a correlation matrix for the Big Five variables). These findings indicate that, although gender differences in guilt-based prosocial behavior are similarly observed among these countries, the cognitive underpinnings may be heterogeneous depending on the social systems, culture, etc.

## Discussion

In this study, we conducted an online behavioral study (Korea, *n* = 294 [175 women]; UK, *n* = 347 [190 women]) of a trust game that was designed to investigate guilt aversion. We found that men exhibited stronger guilt aversion than women in both Korea and the UK Additionally, our Lasso regression, including interaction terms between the gender variable and the Big Five variables, revealed that conscientiousness and neuroticism are key to men’s predominance in guilt aversion in the UK and Korea, respectively.

These results suggest that men’s predominance of guilt aversion may be universal in different cultures but the underlying mechanisms for the gender difference may differ. Guilt aversion includes an aspect of normative behavior elicited by experience (i.e., rule-based decision making^42^). Our current results and our previous study^21^ show that men utilize rule-based strategies more in the UK and Japan. Simultaneously, guilt aversion represents social pressure and may be related to sensitivity to negative emotions^10^. The correlation between men’s predominance in guilt aversion and neuroticism in the Korean population may echo this aspect, suggesting that men in Korea perceive guilt aversion as a strong social pressure.

Previous studies mentioned that online experiments are limited compared with laboratory experimental methods. For example, it is possible that subjects drop out during online experiments, which could jeopardize the experiments’ internal validity^61^. Also, limited attention by the participants in online experiments may hinder the understanding of instructions and cause noise in the results^62^. However, our dropout rate across 45 trials was 8.1% in Korea and 8.0% in the UK, indicating a minimal effect on our results. At the same time, since our participants were not monitored by video camera, we cannot rule out the second problem. Nevertheless, previous studies^63–65^ demonstrated that in online experiments without participant video monitoring, there was no significant difference in social preferences (e.g., fairness and cooperation) compared with laboratory experiments. One of those studies^65^ compared the results of online protocols with and without participant video monitoring with the results of a laboratory protocol for three standard economic games: ultimatum, dictator, and public good, finding only minor differences in the choice data, which indirectly supports the internal validity of our results.

Related to cultural differences, previous research^47,66–68^ found that more gender-equal societies and economically developed countries are associated with larger gender differences in personality traits (Big Five) and social preferences (altruism, trust, reciprocity, risk-taking, and patience). One possible interpretation for this is that people living in more progressive and equal countries have more opportunities to express cultural and biological differences. This view is consistent with the fact that we see men’s predominance of guilt aversion in developed countries (Korea, the UK, and Japan).

Of course, there are alternative explanations for the different contributions of conscientiousness and neuroticism to men’s guilt aversion in Korea and the UK We used the Korean version of The Big Five Inventory-10^51^ translated from the original English version^52^, which was used in our UK experiment. Since this Korean version was designed mainly for the elderly (65 years and older) and the reliability of the scale was tested using elderly subjects (mean age: 73.4 years), the vocabulary familiar to this demographic may be selected in the translation. Therefore, we cannot rule out the possibility that this scale could not measure the Big Five personality traits of younger generations precisely. Second, this short version of Big Five examines five factors with only 10 questions (two questions per factor), which may have made it more difficult to measure the five factors precisely than full versions. Further research is needed to validate the cognitive mechanisms underlying cross-cultural and gender differences in guilt aversion. Nevertheless, our findings about men’s predominance of guilt aversion in Korea, the UK as well as Japan^21^ provide an important clue that helps understand the universality and heterogeneity of gender differences in prosocial behavior.

## Supplementary Information

Supplementary Information.

## Data Availability

The datasets analyzed during the current study are available from the corresponding author upon reasonable request.

## References

[1] Blau FD, Kahn LM (2000). Gender differences in pay. J. Econ. Perspect..

[2] Bertrand M, Ashenfelter O, Card D (2011). New perspectives on gender. Handbook of Labor Economics.

[3] Buser T, Niederle M, Oosterbeek H (2014). Gender, competitiveness, and career choices. Q. J. Econ..

[4] Shurchkov O, Eckel CC (2018). Gender Differences in Behavioral Traits and Labor Market Outcomes.

[5] Boyd CL, Epstein L, Martin AD (2010). Untangling the causal effects of sex on judging. Am. J. Polit. Sci..

[6] Knepper M (2018). When the shadow is the substance: Judge gender and the outcomes of workplace sex discrimination cases. J. Labor Econ..

[7] Tsugawa Y (2017). Comparison of hospital mortality and readmission rates for medicare patients treated by male vs female physicians. JAMA Intern. Med..

[8] Mullola S (2012). Gender differences in teachers’ perceptions of students’ temperament, educational competence, and teachability. Brit. J. Educ. Psychol..

[9] Croson R, Gneezy U (2009). Gender differences in preferences. J. Econ. Lit..

[10] Baumeister RF, Stillwell AM, Heatherton TF (1994). Guilt: An interpersonal approach. Psychol. Bull..

[11] Charness G, Dufwenberg M (2006). Promises and partnership. Econometrica.

[12] Battigalli P, Dufwenberg M (2007). Guilt in games. Am. Econ. Rev..

[13] Battigalli P, Dufwenberg M (2009). Dynamic psychological games. J. Econ. Theory.

[14] Carnì S, Petrocchi N, Del Miglio C, Mancini F, Couyoumdjian A (2013). Intrapsychic and interpersonal guilt: A critical review of the recent literature. Cogn. Process..

[15] Andrighetto G, Grieco D, Tummolini L (2015). Perceived legitimacy of normative expectations motivates compliance with social norms when nobody is watching. Front. Psychol..

[16] Dufwenberg M, Gneezy U (2000). Measuring beliefs in an experimental lost wallet game. Game. Econ. Behav..

[17] Attanasi, G., Battigalli, P. & Nagel, R. Disclosure of belief-dependent preferences in a trust game. *IGIER Working Papers* **506**, (Bocconi University, 2013).

[18] Attanasi G, Battigalli P, Manzoni E, Nagel R (2019). Belief- dependent preferences and reputation: Experimental analysis of a repeated trust game. J. Econ. Behav. Organ..

[19] Battigalli, P. & Dufwenberg, M. Belief-dependent motivations and psychological game theory. *CESifo Working Paper* **8285**. 10.2139/ssrn.3598771 (2020).

[20] Nihonsugi T, Ihara A, Haruno M (2015). Selective increase of intention-based economic decisions by noninvasive brain stimulation to the dorsolateral prefrontal cortex. J. Neurosci..

[21] Nihonsugi T, Numano S, Haruno M (2021). Functional connectivity basis and underlying cognitive mechanisms for gender differences in guilt aversion. ENeuro.

[22] Shoji M (2022). Guilt and prosocial behavior: Lab-in-the-field evidence from Bangladesh. Econ. Dev. Cult. Change.

[23] Reuben E, Sapienza P, Zingales L (2009). Is mistrust self-fulfilling?. Econ. Lett.

[24] Bellemare C, Sebald A, Strobel M (2011). Measuring the willingness to pay to avoid guilt: Estimation using equilibrium and stated belief models. J. Appl. Econom..

[25] Dufwenberg M, Gächter S, Hennig-Schmidt H (2011). The framing of games and the psychology of play. Game. Econ. Behav..

[26] Khalmetski K, Ockenfels A, Werner P (2015). Surprising gifts: Theory and laboratory evidence. J. Econ. Theory.

[27] Khalmetski K (2016). Testing guilt aversion with an exogenous shift in beliefs. Game. Econ. Behav..

[28] Hauge KE (2016). Generosity and guilt: The role of beliefs and moral standards of others. J. Econ. Psychol..

[29] Bellemare C, Sebald A, Suetens S (2018). Heterogeneous guilt sensitivities and incentive effects. Exp. Econ..

[30] Attanasi G, Rimbaud C, Villeval MC (2019). Embezzlement and guilt aversion. J. Econ. Behav. Organ..

[31] Fehr E, Schmidt KM, Kolm SC, Ythier JM (2006). The economics of fairness, reciprocity and altruism? Experimental evidence and new theories. Handbook of the Economics of Giving, Altruism and Reciprocity.

[32] Fehr E, Schmidt KM (1999). A theory of fairness, competition, and cooperation. Q. J. Econ..

[33] Macaskill A, Maltby J, Day L (2002). Forgiveness of self and others and emotional empathy. J. Soc. Psychol..

[34] Toussaint L, Webb JR (2005). Gender differences in the relationship between empathy and forgiveness. J. Soc. Psychol..

[35] Ferguson TJ, Crowley SL (1997). Gender differences in the organization of guilt and shame. Sex Roles.

[36] Benetti-McQuoid J, Bursik K (2005). Individual differences in experiences of and responses to guilt and shame: Examining the lenses of gender and gender role. Sex Roles.

[37] Kugler K, Jones WH (1992). On conceptualizing and assessing guilt. J. Pers. Soc. Psychol..

[38] Jones WH, Schratter AK, Kugler K (2000). The Guilt Inventory. Psychol. Rep..

[39] Else-Quest NM, Higgins A, Allison C, Morton LC (2012). Gender differences in self-conscious emotional experience: A meta-analysis. Psychol. Bull..

[40] Silfver M, Helkama K (2007). Empathy, guilt, and gender: A comparison of two measures of guilt. Scand. J. Psychol..

[41] Hoffman M, Eisenberg N (1982). Development of prosocial motivation: Empathy and guilt. The Development of Prosocial Behavior.

[42] Haidt J, Davidson RJ, Scherer KR, Goldsmith HH (2003). The moral emotions. Handbook of Affective Sciences.

[43] Kleinknecht J (2019). A man of his word? An experiment on gender differences in promise keeping. J. Econ. Behav. Organ..

[44] Chen Y, Zhang Y (2021). Do elicited promises affect people’s trust? Observations in the trust game experiment. J. Behav. Expe. Econ..

[45] World Economic Outlook Database: October 2021. https://www.imf.org/en/Publications/WEO/weo-database/2021/October/download-entire-database (2021).

[46] John OP, Naumann LP, Soto CJ, John OP, Robins RW, Pervin LA (2008). Paradigm shift to the integrative Big Five trait taxonomy: History, measurement, and conceptual issues. Handbook of Personality: Theory and Research.

[47] Costa PT, Terracciano A, McCrae RR (2001). Gender differences in personality traits across cultures: Robust and surprising findings. J. Pers. Soc. Psychol..

[48] Peduzzi P, Concato J, Feinstein AR, Holford TR (1995). Importance of events per independent variable in proportional hazards regression analysis II. Accuracy and precision of regression estimates. J. Clin. Epidemiol..

[49] Ellingsen T, Johannesson M, Tjøtta S, Torsvik G (2010). Testing guilt aversion. Game. Econ. Behav..

[50] Charness G, Samek A, van de Ven J (2022). What is considered deception in experimental economics?. Exp. Econ..

[51] Kim SY (2010). Standardization and validation of big five inventory-Korean version (BFI-K) in elders. Korean J. Biol. Psychiatry.

[52] Rammstedt B, John OP (2007). Measuring personality in one minute or less: A 10-item short version of the Big Five Inventory in English and German. J. Res. Pers..

[53] John OP, Srivastava S, Pervin LA, John OP (1999). The Big Five trait taxonomy: History, measurement, and theoretical perspectives. Handbook of Personality: Theory and Research.

[54] Attanasi G, Battigalli P, Manzoni E (2016). Incomplete-information models of guilt aversion in the trust game. Manage. Sci..

[55] Bracht J, Regner T (2013). Moral emotions and partnership. J. Econ. Psychol..

[56] Bellemare C, Sebald A, Suetens S (2019). Guilt aversion in economics and psychology. J. Econ. Psychol..

[57] Kosmidis, I. *brglm: Bias Reduction in Binary-Response Generalized Linear Models*. https://cran.r-project.org/package=brglm (2019).

[58] Brostrom, G. glmmML: Generalized linear models with clustering. http://cran.r-project.org/web/packages/glmmML/index.html (2008).

[59] Tibshirani R (1996). Regression shrinkage and selection via the lasso. J. R. Stat. Soc B.

[60] Friedman, J. *et al*. Package ‘glmnet’. *CRAN R Repositary* (2021).

[61] Zhou H, Fishbach A (2016). The pitfall of experimenting on the web: How unattended selective attrition leads to surprising (yet false) research conclusions. J. Pers. Soc. Psychol..

[62] Chandler J, Mueller P, Paolacci G (2014). Nonnaı¨vete´ among Amazon Mechanical Turk workers: Consequences and solutions for behavioral researchers. Behav. Res. Methods.

[63] Horton JJ, Rand DG, Zeckhauser RJ (2011). The online laboratory: Conducting experiments in a real labor market. Exp. Econ..

[64] Arechar AA, Gächter S, Molleman L (2018). Conducting interactive experiments online. Exp. Econ..

[65] Buso IM (2021). Lab-like findings from online experiments. J. Econ. Sci. Assoc..

[66] Schmitt DP (2008). Why can't a man be more like a woman? Sex differences in Big Five personality traits across 55 cultures. J. Pers. Soc. Psychol..

[67] Falk A, Hermle J (2018). Relationship of gender differences in preferences to economic development and gender equality. Science.

[68] Giolla EM, Kajonius PJ (2019). Sex differences in personality are larger in gender equal countries: replicating and extending a surprising finding. Int. J. Psychol..

