# Supplementary material for: Gender differences in guilt aversion in Korea and the United Kingdom
Source: Sci Rep. 2022 May 17;12:8187. doi: 10.1038/s41598-022-12163-y (PMC9114390; doi:10.1038/s41598-022-12163-y)
Supplement: Supplementary file 1 — Supplementary Information. [file 41598_2022_12163_MOESM1_ESM.pdf]

Supplementary Information for

Gender differences in guilt aversion in Korea and the United Kingdom

Authors: Tsuyoshi Nihonsugi, Toshiko Tanaka, and Masahiko Haruno

Contents:

Supplemental Results and Discussion

Figure S1. Correlation matrix for the Big Five variables

Figure S2. The results of the Lasso regression of gender differences for inequity

Table S1. Descriptive statistics for the online sample

Table S2. GLM regression of gender differences for guilt

Table S3.GLM regression of gender differences for the cognitive strategy of guilt aversion

Table S4. Lasso regression of gender differences for inequity

Instructions

Personality questionnaire

Supplemental Results and Discussion

Behavioral results of the gender differences for inequity.

For the inequity-aversion behavioral analysis, we replaced the target variable $\beta(Inequity)$ and performed the same Lasso regression as the one for guilt aversion (see Supplementary Fig. S2 and Table S4). Supplementary Fig. S2a and Table S4(1) present the Lasso regression results to identify the relationship between inequity aversion ($\beta(Inequity)$) and gender under a controlled socioeconomic status. We found that

gender had no weight in either the Korean or U.K. populations. However, this result is not consistent with previous empirical studies (for a review, see [1]). For the U.K., openness and education had large positive weights. Supplementary Fig. S2b and Table S4(2) present the Lasso regression results to identify the cognitive mechanisms specific to gender. We found that the interaction term of gender with personality traits had no weight in the Korea or U.K. populations.

Why were there no gender differences for inequity aversion?

Although we found that both countries were inequity averse (see Table 1), we did not observe gender differences in inequity aversion. However, many previous studies have observed differences in inequity aversion, including studies on populations in Korea and the U.K. Those previous studies often used dictator games and social value orientation tasks, which are suitable for focusing on inequity aversion. On the contrary, our trust game was primarily designed to measure guilt aversion. Thus, a potential reason for the inconsistent results is the experimental tasks used. Another potential reason is that the mental concentration of the participants was lower while conducting the task online. Indeed, the withdrawal rate in the present study was higher than in our previous study [2]. In any case, we believe we can remove the effect of inequity aversion from the analysis of guilt aversion using the present task.

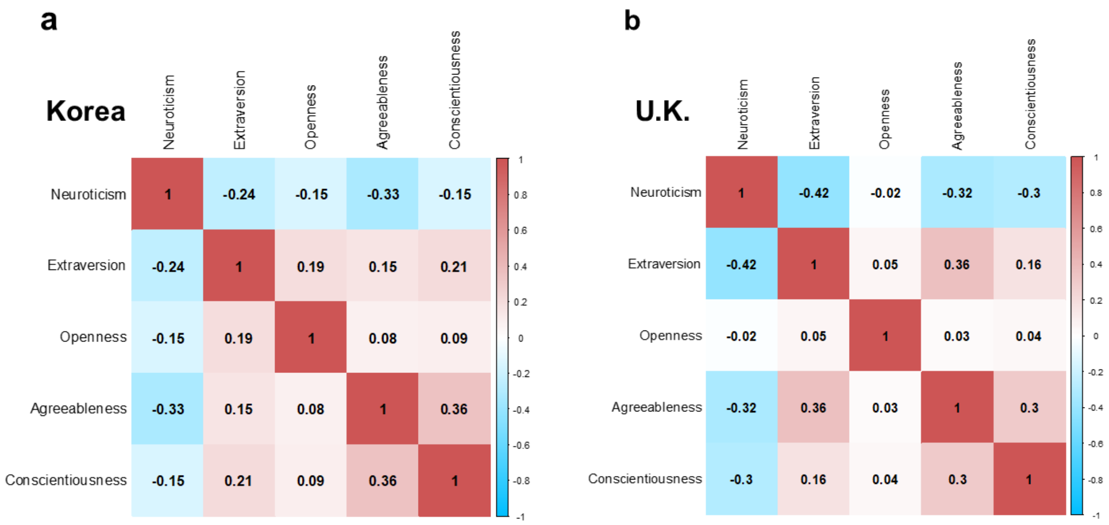

Figure S1. Correlation matrix for the Big Five variables: (a) Korea; (b) U.K.

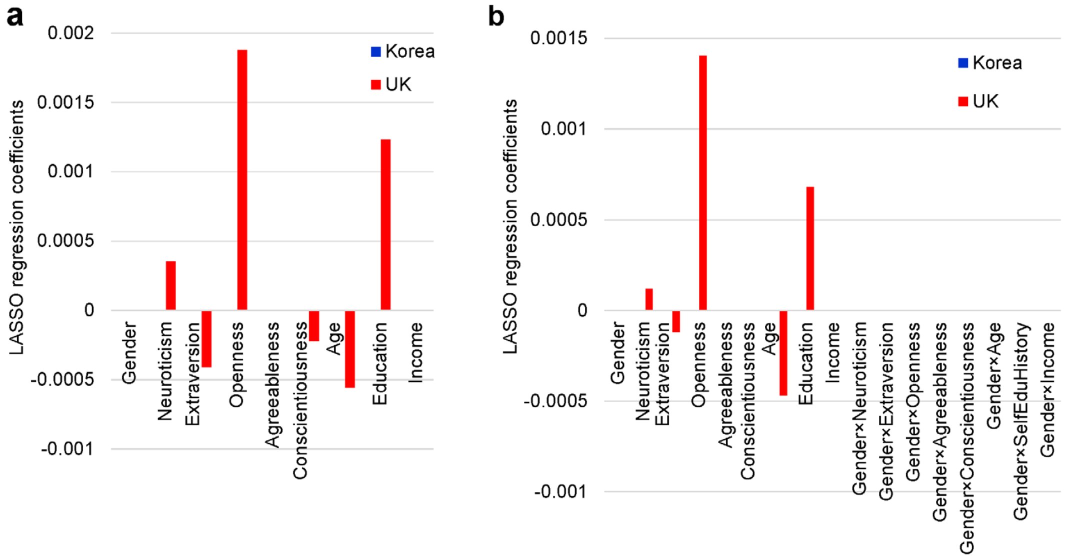

Figure S2. The results of the Lasso regression of gender differences for inequity (except the intercept term for display purposes). Inequity-aversion behavior ($\beta(Inequity)$) was regressed using Lasso regression, with the following variables as regressors: (a) gender, Big Five, and socioeconomic status (age, education, and income); and (b) gender, Big Five, socioeconomic status, and the interaction between gender and Big Five.

Table S1. Descriptive statistics for the online sample

Variables	Korea			U.K.		
	All (n = 294)	Men (n = 119)	Women (n = 175)	All (n = 347)	Men (n = 157)	Women (n = 190)
Neuroticism	5.88	5.39	6.21	6.28	5.69	6.76
	(1.63)	(1.62)	(1.55)	(2.24)	(2.19)	(2.16)
Extraversion	5.96	5.83	6.04	5.61	5.35	5.83
	(1.54)	(1.50)	(1.56)	(204)	(200)	(206)
Openness	6.76	6.31	7.06	7.15	7.10	7.19
	(1.77)	(1.83)	(1.67)	(1.73)	(1.66)	(1.78)
Agreeableness	7.04	7.08	7.01	7.01	6.89	7.11
	(1.31)	(1.42)	(1.24)	(1.81)	(1.87)	(1.77)
Conscientiousness	6.99	7.01	6.97	7.75	7.69	7.80
	(1.39)	(1.46)	(1.35)	(1.62)	(1.68)	(1.57)
Age	35.29	34.82	35.60	37.09	37.72	36.56
	(8.03)	(7.95)	(8.10)	(8.55)	(8.29)	(8.75)
Education	5.65	5.60	5.69	5.10	5.03	5.16
	(0.82)	(0.87)	(0.78)	(1.46)	(1.54)	(1.40)
Income	2.57	2.83	2.39	3.00	3.43	2.64
	(1.20)	(1.28)	(1.11)	(1.4)	(1.44)	(1.25)

Notes: All scores are mean scores. Standard deviations are in parentheses. The Big Five Inventory is a 10-point scale. Education and Income are a 7-point scale.

Table S2. GLM regression of gender differences for guilt

	Dependent variable: $\beta(Guilt)$			
	Korea		U.K.	
Explanatory variable	Model 1	Model 2	Model 1	Model 2
Gender	18.358*	14.818*	16.000**	14.543**
	(8.323)	(7.509)	(5.590)	(5.344)
Neuroticism	1.862		2.540	2.686
	(2.605)		(1.390)	(1.373)
Extraversion	1.372		0.011	-0.162
	(2.589)		(1.440)	(1.423)
Openness	2.878		0.200	
	(2.216)		(1.460)	
Agreeableness	1.606		-1.670	-1.607
	(3.172)		(1.550)	(1.539)
Conscientiousness	-5.950*		2.080	2.122
	(3.002)		(1.670)	(1.657)
Age	0.764		0.108	
	(0.496)		(0.295)	
Education	-0.75		3.200	2.618
	(4.767)		(1.850)	(1.718)
Income	2.438		-2.010	
	(3.444)		(2.050)	
(Intercept)	-11.87		-14.300	
	(45.882)		(28.100)	
Adjusted R^2	0.010	0.010	0.019	0.024
Observations	294	294	347	347

Notes: Standard errors are in parentheses. All coefficients and standard errors are shown multiplied by 10^3. Significance: ** $P < 0.01$; * $P < 0.05$.

Table S3. GLM regression of gender differences for the cognitive strategy of guilt aversion

	Dependent variable: $\beta(Guilt)$			
	Korea		U.K.	
Explanatory variable	Model 1	Model 2	Model 1	Model 2
Gender	-167.182<br>(94.471)		51.140<br>(55.472)	
Neuroticism	-2.395<br>(3.440)		2.940<br>(1.939)	2.493<br>(1.335)
Extraversion	0.467<br>(3.360)		-0.926<br>(1.898)	-0.222<br>(1.411)
Openness	2.402<br>(2.960)		1.041<br>(1.943)	
Agreeableness	-4.135<br>(4.558)		0.156<br>(2.129)	-1.363<br>(1.493)
Conscientiousness	-2.244<br>(4.152)		-0.200<br>(2.374)	
Age	0.483<br>(0.630)		0.380<br>(0.392)	
Education	-3.818<br>(6.490)		2.861<br>(2.601)	2.661<br>(1.715)
Income	4.954<br>(4.467)		-0.294<br>(2.890)	
Gender × Neuroticism	10.098<br>(5.539)	3.083*<br>(1.296)	-1.326<br>(2.815)	
Gender × Extraversion	3.245<br>(5.453)		1.907<br>(2.964)	
Gender × Openness	2.521<br>(4.550)		-2.151<br>(3.003)	
Gender × Agreeableness	13.334*<br>(6.504)		-4.038<br>(3.140)	
Gender × Conscientiousness	-5.407<br>(6.192)		4.728<br>(3.390)	1.998**<br>(0.663)
Gender × Age	0.491<br>(1.098)		-0.630<br>(0.602)	
Gender × Education	7.860<br>(9.738)		0.654<br>(3.741)	
Gender × Income	-9.432<br>(7.540)		-3.523<br>(4.137)	
(Intercept)	59.221<br>(60.846)		-25.428<br>(39.375)	
Adjusted R^2	0.010	0.018	0.011	0.029
Observations	294	294	347	347

Notes: Standard errors are in parentheses. All coefficients and standard errors are shown multiplied by 10^3. Significance: ** $P < 0.01$; * $P < 0.05$.

Table S4. Lasso regression of gender differences for inequity

	Dependent variable: $\beta(Inequity)$			
	(1)		(2)	
Explanatory variable	Korea	U.K.	Korea	U.K.
Gender	0	0	0	0
Neuroticism	0	0.351	0	0.121
Extraversion	0	−0.408	0	−0.120
Openness	0	1.877	0	1.405
Agreeableness	0	0	0	0
Conscientiousness	0	−0.223	0	0
Age	0	−0.558	0	−0.469
Education	0	1.234	0	0.681
Income	0	0	0	0
Gender × Neuroticism			0	0
Gender × Extraversion			0	0
Gender × Openness			0	0
Gender × Agreeableness			0	0
Gender × Conscientiousness			0	0
Gender × Age			0	0
Gender × Education			0	0
Gender × Income			0	0
(Intercept)	13.785	20.844	13.785	21.852

Notes: All scores are Lasso regression coefficients and are shown multiplied by 10^3.

Instructions

1. General introduction

In this experiment, you will be paired with another person. In each pair, one person will be Player A and the other will be Player B. You will be participating in a game, which will be explained later. During this experiment, you will be asked to make a number of decisions, and you will change pairs each time you make a decision so that you are paired with the same person only once. The experiment is strictly anonymous; that is, your identity will not be revealed to others, and others' identities will not be revealed to you.

2. Rules of the game

Please review and ensure that you understand the rules of the game by referring to the following figure. Every time you make a decision, a figure similar to the one below will be displayed, but the values in the figure will change.

1. In the first stage, Player A will make two decisions based on the amount of money in the game. For the first decision, Player A must choose W or Z. If Player A chooses Z, Players A and B will receive z_A and z_B pence, respectively. If Player A chooses W, then Player B will make the decision.

   For the second decision, Player A must report a belief probability from 0% to 100% in increments of 10% that Player B (partner) will choose R.

2. If Player A chooses W, then given Player A's belief probability, Player B must choose L or R. For example, if Player B chooses L, Player A receives y_A pence and Player B receives y_B pence; if Player B chooses R, then Players A and B will receive x_A and x_B pence, respectively.

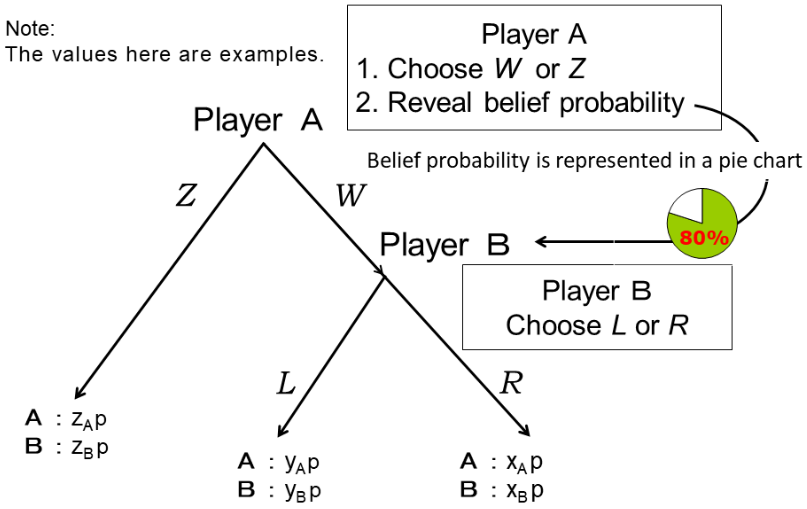

3. Procedure of the experiment

Here, we will explain the tasks along the flow of the experiment. You will first make one decision as Player A and then make 45 further decisions as Player B.

1. First, a game will be displayed on your screen, and you (as Player A) will choose *W* or *Z* and reveal your belief probability that Player B will choose *R* based on the amount of money in the game. You will experience one trial as Player A. Your choices will be used when Player B makes their choice in the next stage.

   In this experiment, many participants will make decisions as Player A, just as you did; therefore, it is uncertain whether the decision you make as Player A will be used in the next stage. If your choice is used in the next stage, you will receive money according to the choices made by both you and your partner. Therefore, if you choose *Z* in today's experiment and the earnings are already fixed, you will not be paid if it is not used in the experiment. We will contact you again if we have to pay.

2. Next, another game will be displayed on your screen. In this game, you will make decisions as Player B. Your partner (Player A) is one of the many participants. Player A has already made a decision, but without knowing Player A's choice, you assume that Player A has chosen *W* and make your own decision. Please choose *L* or *R* based on the amount of money and Player A's belief probability that you will choose *R*. You will play the game as Player B for 45 trials. Each time you make a decision, you will have a new

Player A, and the amount of money and the belief probability will also change. After 45 decisions, the experiment is complete.

Finally, your earnings will be equal to the amount of money you earned from the one decision you made as Player A and the 45 decisions you made as Player B multiplied by 0.07. Since your decisions directly affect you and your partner's earnings, please concentrate on the task during the experiment. This concludes the instructions.

Personality questionnaire

(This case was used in the U.K. experiment)

Questions	Options
Age	(free description)
Gender	･Female<br>･Male
Select the option that closely represents your personal educational history. Exclude any currently enrolled schooling.	･Primary school graduate<br>･Secondary school graduate or similar<br>･High School/Sixth form/Tertiary/Tech. College dropout (attended at least 1 year) or similar<br>･High School/Sixth form/Tertiary/Tech. College graduate or similar<br>･University dropout/ junior college (2-year program) or similar graduate or dropout (attended at least 1 year)<br>･Bachelor's degree<br>･Master's degree or higher
Select your annual income from the options listed.	･£0–£10,000<br>･£10,000–£20,000<br>･£20,000–£35,000<br>･£35,000–£55,000<br>･£55,000–£70,000<br>･£70,000–£100,000<br>･more than £100,000
How well do the following statements describe your personality?<br>I see myself as someone who…	
is reserved	1. Disagree strongly<br>2. Disagree a little<br>3. Neither agree nor disagree<br>4. Agree a little<br>5. Agree strongly

is generally trusting	1. Disagree strongly<br>2. Disagree a little<br>3. Neither agree nor disagree<br>4. Agree a little<br>5. Agree strongly
tends to be lazy	1. Disagree strongly<br>2. Disagree a little<br>3. Neither agree nor disagree<br>4. Agree a little<br>5. Agree strongly
is relaxed, handles stress well	1. Disagree strongly<br>2. Disagree a little<br>3. Neither agree nor disagree<br>4. Agree a little<br>5. Agree strongly
has few artistic interests	1. Disagree strongly<br>2. Disagree a little<br>3. Neither agree nor disagree<br>4. Agree a little<br>5. Agree strongly
is outgoing, sociable	1. Disagree strongly<br>2. Disagree a little<br>3. Neither agree nor disagree<br>4. Agree a little<br>5. Agree strongly
tends to find fault with others	1. Disagree strongly<br>2. Disagree a little<br>3. Neither agree nor disagree<br>4. Agree a little<br>5. Agree strongly
does a thorough job	1. Disagree strongly<br>2. Disagree a little<br>3. Neither agree nor disagree<br>4. Agree a little<br>5. Agree strongly
gets nervous easily	1. Disagree strongly

	2. Disagree a little<br>3. Neither agree nor disagree<br>4. Agree a little<br>5. Agree strongly
has an active imagination	1. Disagree strongly<br>2. Disagree a little<br>3. Neither agree nor disagree<br>4. Agree a little<br>5. Agree strongly

References

1. Croson, R. & Gneezy, U. Gender differences in preferences. *J. Econ. Lit.* **47**, 448-474 (2009).

2. Nihonsugi, T., Numano, S. & Haruno M. Functional connectivity basis and underlying cognitive mechanisms for gender differences in guilt aversion. *eNeuro*, https://doi.org/10.1523/ENEURO.0226-21.2021 (2021).
